# Autoimmune Hematologic Disorders in Two Patients After mRNA COVID-19 Vaccine

**DOI:** 10.1097/HS9.0000000000000618

**Published:** 2021-07-13

**Authors:** Marie-Estelle Gaignard, Sven Lieberherr, Andreas Schoenenberger, Rudolf Benz

**Affiliations:** 1 Department of Internal Medicine, Kantonsspital Muensterlingen, Switzerland; 2 Department of Hematology and Oncology, Kantonsspital Muensterlingen, Switzerland

Since the occurrence of SARS-CoV-2 infections in late 2019, >3.4 million people died of COVID-19 worldwide and many more suffered from its severe complications and long-term residual symptoms. Autoimmune hematologic disorders such as immune thrombocytopenia (ITP) or autoimmune hemolytic anemia (AIHA) have been reported as complications of COVID-19.^1,2^ Due to ITP manifestations in asymptomatic COVID-19 patients, these cases likely underestimate their real occurrence.^1,3^ Some authors even postulated a causal relationship between a prothrombotic state in ITP patients and SARS-CoV-2 infections, leading to a highly delicate and challenging situation.^4^ Similarly, AIHA is a probably underrated complication of COVID-19, and Algassim et al^5^ reported that lower hemoglobin levels are related to poorer prognosis in these patients. These complications therefore represent an additional burden of a disease whose potentially severe clinical course mainly affects elderly patients. Fortunately, vaccination campaigns against COVID-19 are now well underway throughout Europe and around the globe. Like any vaccine, COVID-19 vaccines have been associated with mild-to-moderate side effects, as pain at the injection site, fever, or muscle pain. Recently, concerns have also been raised about serious side effects associated with the AZD1222 vaccine, including an increased risk of sinus vein thrombosis. An immunologically mediated HIT-like mechanism via antibodies against PF4 is postulated as the underlying pathophysiologic mechanism.^6^ Likewise, a few dozen cases of classical ITP manifestations after vaccination against COVID-19 have been reported in recent weeks.^7–11^ Vaccine-related autoimmune hematologic disorders, especially ITP, are well known and can typically be seen after MMR vaccinations.^12^ Due to the much lower incidence of such phenomena after vaccination compared to postnatural infections, their mainly mild clinical course as well as their many other significant benefits, these vaccines are still highly recommended. Here, we report two different cases of autoimmune hematologic disorders, ITP and AIHA, respectively, occurring with a clear timely correlation after administration of Moderna mRNA-1273 COVID-19 vaccine. These cases were already reported to the national drug regulatory authority Swissmedic via the electronic pharmacovigilance reporting system.

A 56-year-old male was admitted to the emergency department with unprovoked multiple painless petechia located on the oral mucous membranes of the lower lip and to a smaller extent on the skin of his left upper arm where he reported receiving his first injection of the Moderna COVID-19 vaccine 3 days ago (Figure 1). His medical history included Evans Syndrome, which was diagnosed in 2015. In 2011, the patient initially presented with an isolated severe thrombocytopenia (2 × 10^9^/L) while showing a normal hemoglobin level (169 g/L). At the time of diagnosis, a peripheral blood smear revealed single giant platelets, while bone marrow cytology and flowcytometry detected no signs of an underlying hematologic disorder. The complete workup did not find any secondary causes associated with ITP. The patient was back then treated with 250 mg prednisolone and a cumulative dose of 80 g intravenous immunoglobulin (IVIg) followed by 100 mg prednisolone for 20 days. Due to insufficient rise of thrombocytes, a 4-day dexamethasone 40 mg pulse therapy followed by steroid tapering was performed. His platelet count normalized rapidly. In 2015, he developed a severe normochromic normocytic anemia (hemoglobin 74.2 g/L), with warm autoantibodies detected in the direct antiglobulin test, leading to the diagnosis of Evans syndrome. The patient received an 80 mg methylprednisolone pulse therapy with subsequent tapering. The first time we saw him in our clinic was in 2019 due to a relapse of his ITP with a minimal platelet count of 2 × 10^9^/L. The patient was then successfully treated with 40 mg dexamethasone pulse therapy for 4 days and 90 mg IVIg followed by steroid tapering. Thrombocyte levels have been normal since then. The patient additionally had a history of autosomal dominant polycystic kidney disease with hypertension. His current medical treatment consists of antihypertensive drugs and aspirin, which had been initiated after a dissection of the renal artery 2 years ago. On the current admission, no major bleeding occurred. A sonography of the abdomen showed a slight splenomegaly (diameter 15 cm). Laboratory findings revealed a severe thrombocytopenia (3 × 10^9^/L) with normal hemoglobin (151 g/L) and leukocyte (6.6 × 10^9^/L) counts. PTT, fibrinogen, and D-dimer levels were also normal. Microscopic evaluation of a peripheral blood smear confirmed the absence of pseudothrombocytopenia or other hematological disorders. No schistocytes were detected. Due to his known medical condition, the diagnosis of ITP relapse was made. Aspirin was discontinued and a 4-day course of 40 mg dexamethasone daily was started with rapid remission of clinical lesions. At day 2, 1 g/kg IVIg was administered. However, because of the lack of increase in thrombocytes, the corticosteroid therapy was continued with prednisone 1 mg/kg and a thrombopoietin receptor agonist (TPO-RA) was initiated at day 7. After a total of 2 repeated IVIg infusions, daily prednisone and weekly TPO-RA administrations, platelet counts normalized within 2 weeks and the patient could be discharged (Figure 2). Prednisone was then tapered. Due to a platelet count above 400 × 10^9^/L, the TPO-RA was stopped after 3 injections of a maximal dose of 125 µg romiplostim weekly. At 3-month follow-up, the patient was relapse-free and off any ITP treatment and no thrombosis occurred.

**Figure 1.**
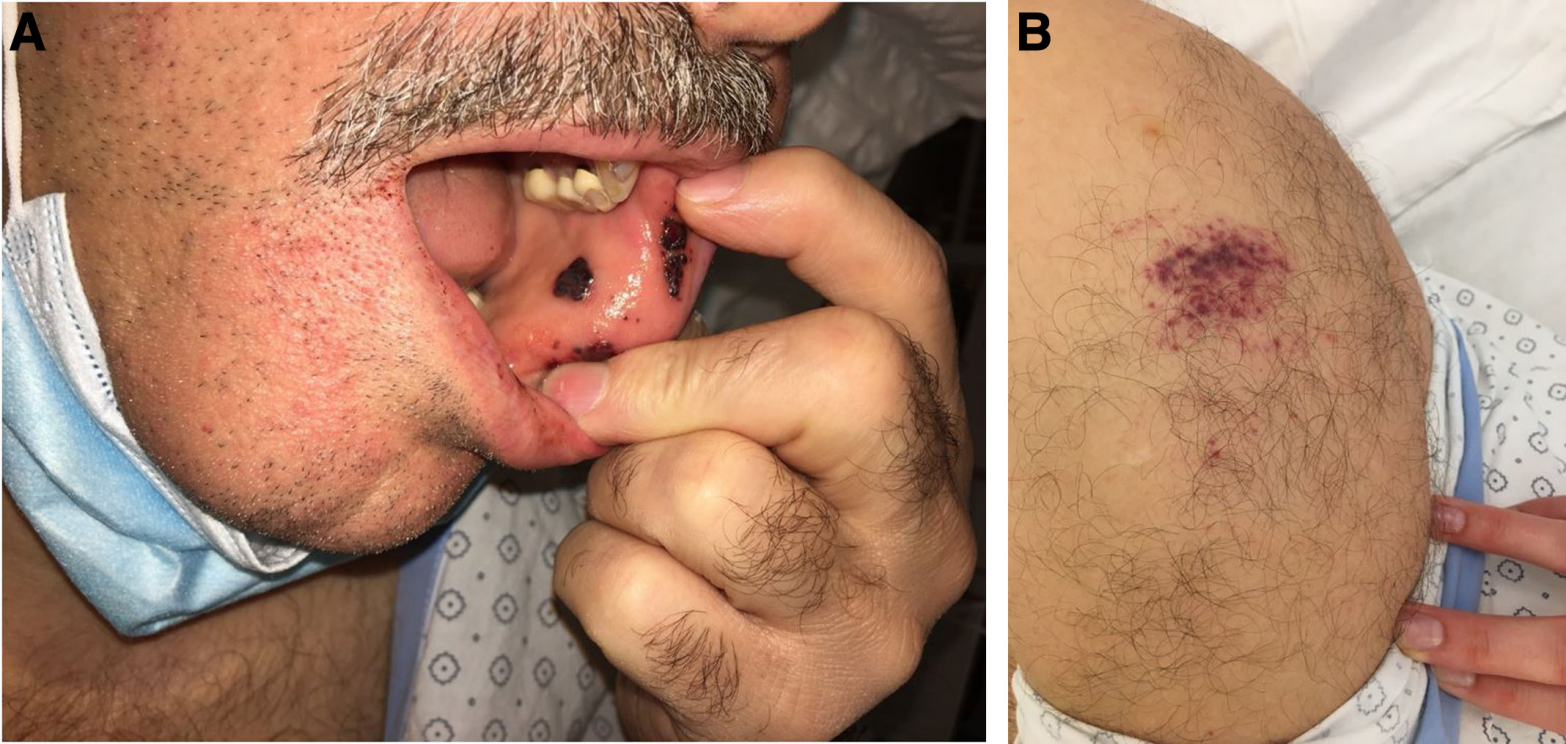
Mucosal purpura on the inner lower lip and multiple cutaneous petechiae at the vaccine injection site on the left upper arm (deltoid region).

**Figure 2.**
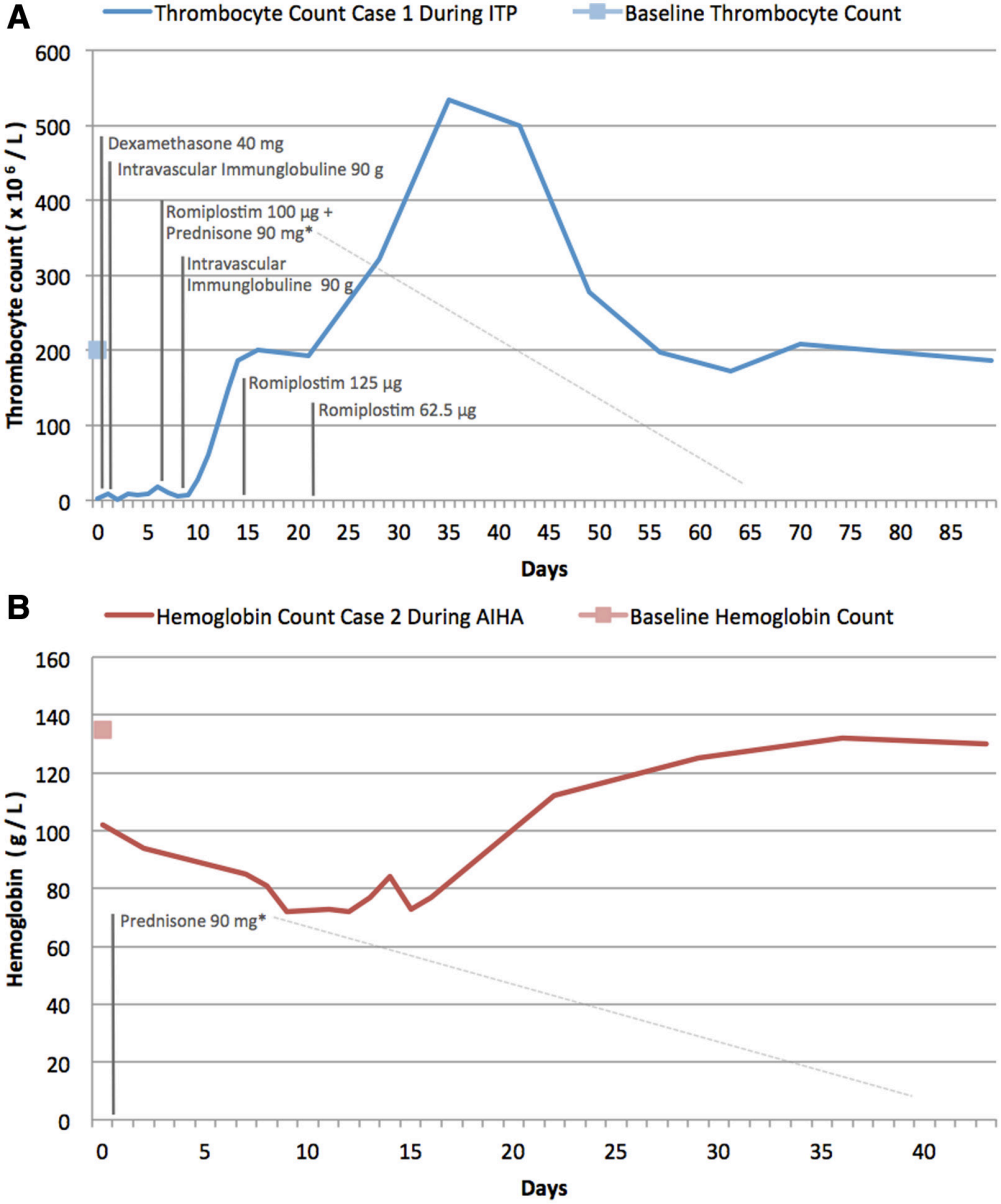
**Course of selected laboratory parameters under treatment.** Variation in absolute platelet (A) and hemoglobin (B) count over time. *Steroid tapering.

A healthy 77-year-old man without known comorbidities and with no medication use received his first Moderna mRNA-1273 vaccine in the context of the regional vaccination campaign. On day 5 postvaccination, he experienced weakness, fatigue and shortness of breath which led him to consult his general practitioner. Due to a newly diagnosed anemia the patient was referred to our clinic. Well-being until vaccination and no history of a recent infection were reported. Unlike in case 1, no personal or family history of bleeding or autoimmune disease was noted. His vital signs and physical examination were normal and chest radiography showed no abnormality. A complete blood count revealed a normochromic normocytic anemia (86 g/L) with increased reticulocytes (310 × 10^9^/l), mild leukocytosis (11.7 × 10^9^/L) with an unremarkable differential blood count and platelets of 344 × 10^9^/L. Laboratory tests also indicated elevated transaminases, LDH and bilirubin and a decreased haptoglobin. Abdominal ultrasound showed discrete inhomogeneous liver parenchyma. Further virological laboratory tests for HIV, hepatitis B, C, A, and E were performed, which, except for positive HEV–IgG, turned out negative. A HEV-RT-PCR test was realized accordingly and proved negative. Antimyeloperoxidase (MPO), p-ANCA, c-ANCA antibody levels were also measured and found to be within normal range. Due to the concomitant presence of a high LDH value with a low haptoglobin, the patient was screened for IAT, IgG- and C3- DAT, which were all positive. Warm AIHA was diagnosed. To rule out lymphoma, a chest-abdomen CT scan and a bone marrow examination were performed and showed no abnormality. The protein electrophoresis was also normal. We started a corticosteroid therapy with prednisone 75 mg. Two weeks later, his hemoglobin count increased to 112 g/L (Figure 2). Reticulocytes, bilirubin, and LDH levels also decreased. Prednisone could be gradually tapered and the patient’s hemoglobin level has returned to almost normal after 10 weeks.

To prevent not only a potentially severe clinical course of the infection, but also its harmful associated conditions, vaccination offers to date the best option to successfully overcome the COVID-19 pandemic. Nonetheless, side effects of COVID-19 vaccines have to be recognized and investigated, especially if they involve therapeutic measures that must be implemented rapidly, as in the case of immunologically mediated cytopenias. In addition, some of them can in part be foreseen, if underlying conditions are specifically taken into consideration. As shown in our cases, episodes of disease activity can be triggered by the immune stimulation induced by vaccines. Pathophysiological mechanisms underlying these reactions are related to molecular mimicry, epitope spreading, and polyclonal activation.^13^ Because classical immunostimulatory adjuvants are not part of mRNA vaccines and the involved antigens in ITP and AIHA are different, novel immunostimulatory mechanism seem more plausible.

The management of these vaccine-related ITP and AIHA should be similar to that used in primary conditions, that is, by using steroids, IVIg and, for ITP, TPO receptor agonists. If these disorders occur after first vaccination, we believe that the second dose should at least be postponed. While there is no general consensus yet on a level of antibody protection against the S-antigen of the virus, the test can help to discuss the risks and benefits of a second vaccination. However, rituximab, which can be used as a second- or third-line treatment for AIHA and ITP, should be employed with caution on a case-by-case basis because of the significant impairment of the humoral immune response associated with it.^12^ The question of whether TPO-RA should be used in the treatment of ITP also remains a matter of debate. To date, there seems to be no firm evidence of increased thrombosis rates in mRNA-based COVID-19 vaccines compared with AZD1222-related cases. However, risks and benefits should be weighed with care and tailored to each patient and situation.

In conclusion, autoimmune hematological disorders after COVID-19 vaccines can be observed in patients with preexisting conditions as well as in healthy individuals. Disease course seems, however, to be mild and timely limited. The benefits of these vaccines still by far outweigh the risks, even in patients with known underlying autoimmune hematologic disease. Nonetheless, awareness on their potentially serious side effects and their management is key.

## Disclosures

The authors have no conflicts of interest to disclose.
